# Human Pose Estimation Based on Efficient and Lightweight High-Resolution Network (EL-HRNet)

**DOI:** 10.3390/s24020396

**Published:** 2024-01-09

**Authors:** Rui Li, An Yan, Shiqiang Yang, Duo He, Xin Zeng, Hongyan Liu

**Affiliations:** 1 School of Mechanical and Precision Instrument Engineering, Xi’an University of Technology, Xi’an 710054, China; liruizixing@163.com (R.L.); 2210221204@stu.xaut.edu.cn (A.Y.); heduo98@163.com (D.H.); 2210221215@stu.xaut.edu.cn (X.Z.); liuhy_1@163.com (H.L.); 2 Xi’an People’s Hospital, Xi’an 710054, China

**Keywords:** human pose estimation, lightweight network, HRNet, CBAM

## Abstract

As an important direction in computer vision, human pose estimation has received extensive attention in recent years. A High-Resolution Network (HRNet) can achieve effective estimation results as a classical human pose estimation method. However, the complex structure of the model is not conducive to deployment under limited computer resources. Therefore, an improved Efficient and Lightweight HRNet (EL-HRNet) model is proposed. In detail, point-wise and grouped convolutions were used to construct a lightweight residual module, replacing the original 3 × 3 module to reduce the parameters. To compensate for the information loss caused by the network’s lightweight nature, the Convolutional Block Attention Module (CBAM) is introduced after the new lightweight residual module to construct the Lightweight Attention Basicblock (LA-Basicblock) module to achieve high-precision human pose estimation. To verify the effectiveness of the proposed EL-HRNet, experiments were carried out using the COCO2017 and MPII datasets. The experimental results show that the EL-HRNet model requires only 5 million parameters and 2.0 GFlops calculations and achieves an AP score of 67.1% on the COCO2017 validation set. In addition, PCKh@0.5mean is 87.7% on the MPII validation set, and EL-HRNet shows a good balance between model complexity and human pose estimation accuracy.

## 1. Introduction

The pose is one of the important biological characteristics of the human body, and human pose estimation aims to detect the keypoints of the human body in pictures or videos to describe the human pose. It is an important research direction in computer vision and the basis for computer understanding of human actions and behaviors and has important research significance for realistic video surveillance, human–computer interaction, medical rehabilitation, intelligent driving, and other typical application scenarios [1,2,3].

Traditional methods for human pose estimation mainly use graph-structure-based models [4,5], which rely on hand-designed features, have poor robustness, and are not suitable for practical applications. Deep-learning algorithms have made a splash in the field of computing because of their excellent learning capabilities, and researchers have focused on how to implement human pose estimation tasks using deep learning.

DeepPose [6] first applied a deep neural network to human body pose estimation, planned the pose estimation task as a regression problem based on a deep neural network to detect the keypoints of the body, and obtained good pose estimation results. Subsequently, based on the Cascaded Pyramid Network (CPN) [7], Multi-Stage Pose Network (MSPN) [8], Residual Steps Network (RSN) [9], and other deep-learning methods have emerged for solving the problems of keypoint occlusion, environmental interference, and complex backgrounds in pose estimation. For example, Kan [10] et al. divided the body keypoints into six structural groups, each of which was further divided into terminal and base keypoints. This group further developed a self-constrained prediction–validation network to learn the structural correlations between these two subsets within each structural group. Although the accuracy of the human pose estimation algorithm has achieved good results, with the development of human pose estimation, the algorithm structure used for pose estimation is becoming more and more complex, such as HRNet [11], HigherHRNet [12], TransPose [13], ViTPose [14], and other networks. Although these methods can achieve high-precision human pose estimation, the convolutional layer of the network is getting deeper and deeper, and the number of parameters and computations is also rising, which makes related experiments require increasingly higher computer equipment performance, which is not conducive to the practical application of human pose estimation. It is difficult to achieve accurate pose estimation when the background color is cluttered and complex, the body parts are occluded, or the body color is similar to the surrounding environment. Maintaining high-resolution information is very important for the detection of these keypoints. However, in each network structure that maintains high-resolution information, there is high network complexity and a large number of calculation parameters. Therefore, a major challenge in pose estimation is how to have fewer parameters and better performance while preserving high-resolution information. Among them, HRNet achieves high accuracy in the task of human pose estimation, but its parameter number and computational complexity are high. Thus, lightening the network is a major challenge in the field of pose estimation. It is challenging to balance the complexity and accuracy of the model because of the loss of accuracy caused by the light weight of the model.

To reduce the computational power and memory requirements of the computer while maintaining the accuracy of pose estimation, some scholars [15,16,17,18] have researched human posture estimation methods based on lightweight models. Still, the existing methods cannot maintain a good balance between the model complexity and the accuracy of human posture estimation. This paper focuses on reducing the demand for efficient human pose estimation models on device memory and computational resources to achieve lightweight models while maintaining their performance in human pose estimation tasks.

HRNet can achieve high accuracy in human pose estimation tasks, but maintaining high-resolution representation also increases the number of model parameters and the computational burden. Therefore, this paper aims to solve the conflict between high-resolution representation and lightweight models by using HRNet as the object. The main body of HRNet contains four feature extraction stages, the last three of which are performed by Basicblock [19] modules, and therefore, Basicblock occupies a large proportion of the structure of the HRNet network. In this work, we conducted a lightweight study on the Basicblock structure and firstly constructed a lightweight Basicblock module (L-Basicblock, Lightweight Basicblock), but this approach caused the loss of feature information in the human pose estimation process.

Attention mechanisms [20,21] can obtain the target area that needs to be focused on and then obtain target detail information from that area and suppress useless information. Many excellent attention mechanisms have emerged in recent years [20,22,23,24,25] that can be easily applied to the pose estimation network to extract critical human information. Therefore, in this paper, to compensate for the information loss in the network caused by the module’s lightweight nature, the CBAM attention mechanism is further introduced in L-Basicblock [26], and then, the LA-Basicblock module is constructed to propose a lightweight and effective human pose estimation model named EL-HRNet; the model can maintain its performance while achieving a lightweight network.

The main contributions of this work can be summarized as follows:

(1) To solve the problem of the high number of parameters and computations of the pose estimation network HRNet, the Basicblock module, which is widely used in HRNet, is improved, and a lightweight L-Basicblock module is built to reduce the number of parameters and computations of the model and speed up the output of human pose estimation results;
(2) To improve the problem of feature information loss caused by the module’s lightweight nature, the CBAM attention mechanism is further introduced in L-Basicblock, and finally, the LA-Basicblock module is constructed to pay attention to the feature information of different resolution subnetworks in HRNet to obtain rich, effective human keypoint information and suppress invalid information;
(3) A lightweight human pose estimation model, EL-HRNet, with an excellent balance of complexity and accuracy, is constructed to achieve an accurate estimation of human pose.

## 2. Materials and Methods

Human pose estimation first detects the representative human keypoints in the picture and then connects the keypoints to form the corresponding limbs to obtain the complete human pose in the picture [11]. High-resolution feature maps contain a lot of effective human keypoint location and semantic information, and obtaining high-resolution feature maps in the process of image processing in human pose estimation models is an effective means to improve the accuracy of human pose estimation. HRNet obtains excellent human pose estimation performance by maintaining high-resolution representations throughout the model and fusing the information of feature maps that have different resolutions through parallel branching, but the increase in high-resolution representations leads to a consequent increase in the number of model parameters and computational effort [17].

In this paper, based on the HRNet model, human pose estimation is studied. Given the problems of a large parameter number and calculation amount, the L-Basicblock module is proposed to reduce the parameter number and calculation amount of the model and reduce the requirements of the model for computer memory and running computing power. Using the combination of a 1 × 1 convolution and 3 × 3 group convolution (GConv), the feature graph is compressed to greatly reduce the number of parameters and computations while maintaining features with different resolutions. Secondly, given the loss of key information about the human body in the image features caused by the light weight of the model, the lightweight CBAM attention mechanism is further introduced based on the L-Basicblock module to build the LA-Basicblock module. The CBAM module can model the key information in the feature map from the channel domain and spatial domain, realizing the dual attention of channel and spatial information, thus improving the ability of the network to represent the human posture. This implements a lightweight and efficient EL-HRNet human pose estimation model. Finally, the improved EL-HRNet human pose estimation model was trained, verified, and tested on two large datasets, COCO [27] and MPII [28], and the OKS (Object Keypoint Similarity) evaluation index was used on the coco dataset. The PCKh (head-normalized probability of correct keypoint) evaluation index is used on the MPII dataset.

### 2.1. HRNet Model

**Figure 1:**
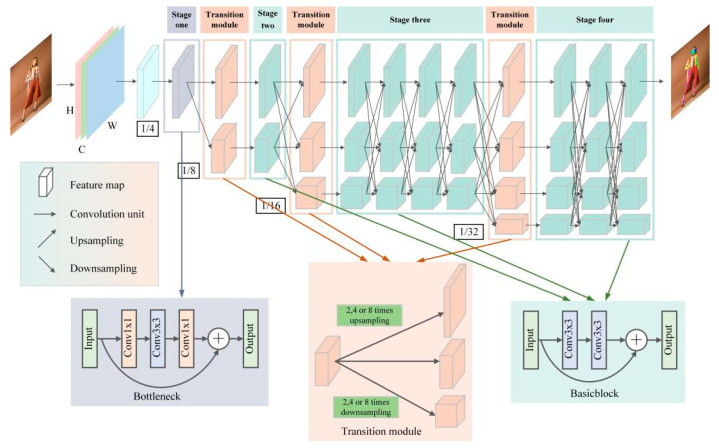
HRNet framework.

HRNet is a classical heatmap-based method for human pose estimation. A picture $I\in {\mathbb{R}}^{W\times H\times C}$ ( W is the width of the picture, H is the height of the picture, C is the channel of the picture, and the input picture has three channels: red, green, and blue) containing the human body is input into the HRNet network, and then a series of convolution operations are performed; finally, the model will output a heatmap $H\in {\mathbb{R}}^{W^{\prime }\times H^{\prime }}$ with *p* human keypoints, and the coordinates with the highest heat value in each heatmap will be scaled to the input picture space. The framework of HRNet is shown in Figure 1.

HRNet contains a total of four feature extraction stages, each of which adds a parallel subnetwork compared to the previous stage, except for the first stage. The first feature extraction stage consists of four Bottleneck modules of width 64 (Figure 1) that compress and then amplify the feature information by a 1 × 1 convolution, 3 × 3 convolution, and 1 × 1 convolution to effectively obtain the underlying features of the input image. The second, third, and fourth feature extraction stages consist of 1, 4, and 3 information exchange modules, respectively, and each information exchange module consists of 4 Basicblock (Figure 1) units; Basicblock learns the feature information with two regular 3 × 3 convolutions. To enable each subnetwork to repeatedly receive multiscale keypoint information from other parallel subnetworks, HRNet introduces transition modules between every two feature extraction stages. Each transition module can implement information transfer by up-sampling or down-sampling. 

When the image containing the human body is input into the pose estimation network, HRNet first reduces the resolution of the input image I to 1/4 through two 3 × 3 convolution steps of 2 to obtain a high-resolution output feature map and keeps the high-resolution representation of the first subnetwork. The resolutions of the feature maps of the second, third, and fourth subnetworks are 1/8, 1/16, and 1/32 of the input image I, respectively. Through the transition module between various stages of HRNet, the keypoint information of the human body in feature maps at different scales can be obtained to improve the accuracy of the human body pose estimation.

In the heatmap regression method, it is necessary to convert the labeled data into training labels to obtain *p* heatmaps $\left\{h_{1},h_{2},\ldots ,h_{p}\right\}$ , with each heatmap having the size w×h . For a particular labeled feature point $u_{i}=(x_{i},y_{i})$ , the value of the feature point ( x,y ) on the real heatmap corresponding to that feature point is as follows:

(1) $$h_{i}(x,y)=e^{-\frac{{\left(x-x_{i}\right)}^{2}+{\left(y-y_{i}\right)}^{2}}{2\sigma^{2}}}\sigma$$

The true heatmap is a Gaussian distribution centered on the feature point $u_{i}$ , where σ is the standard deviation of the manual design, taken as σ = 2.

The loss function of the human pose estimation network is defined as the Mean Square Error (*MSE*), which is used to compare the predicted heatmap with the real heatmap, and the result obtained by the human pose estimation network is the predicted heatmap. Specifically, for the position of each joint, the difference between its estimated value and the true value is squared, and the average value is taken as the value of the loss function. The loss function *MSE* is defined as follows:

(2) $$MSE=\frac{{\sum_{i=1}^{p}\sum_{j=1}^{w}\sum_{k=1}^{h}\left[h_{i}\left(x,y\right)-\hat{h_{i}}\left(x,y\right)\right]}^{2}}{p\cdot w\cdot h}$$

where *w* and *h* are the width and height of the predicted heatmap (one-quarter of the input image size *W* and *H*), *p* is the number of keypoints, and $h_{i}(x,y)$ and ${\hat{h}}_{i}(x,y)$ are the coordinates of the keypoints in the real heatmap and the predicted heatmap, respectively. 

### 2.2. HRNet Model with the Introduction of a Lightweight Residual Module

#### 2.2.1. Building the Lightweight Residual Module

**Figure 2:**
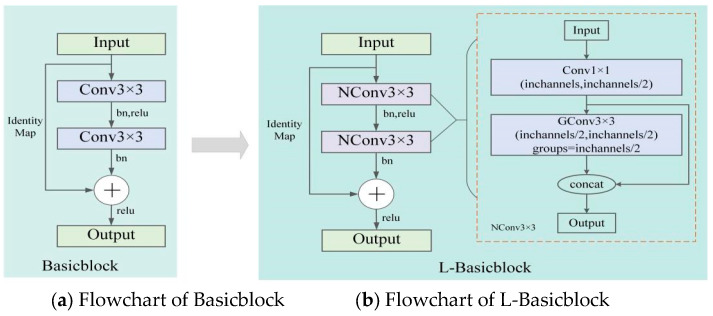
Flowchart of Basicblock and L-Basicblock. (**a**) Flowchart of Basicblock; (**b**) Flowchart of L-Basicblock.

In the HRNet human pose estimation model, Basicblock is used in the second, third, and fourth stages, while the Bottleneck module is used in the first feature extraction stage, which has the advantage of an additional short-cut branch compared with the traditional convolutional structure to channel the input information directly to the output, which can solve the training difficulties caused by the increasing depth of the network [18]. The original Basicblock structure is shown in Figure 2a, and its main structure consists of two 3 × 3 convolutions, so this structure leads to a large number of parameters and computations of the HRNet network.

To reduce the number of parameters and computations of the HRNet, this study constructed a module called Lightweight Basicblock (L-Basicblock) based on the Basicblock module by proposing a new convolution structure (NConv3 × 3, New Convolution with 3 × 3 kernel size). The structure of NConv3 × 3 is shown in Figure 2b, which is a combination of a 1 × 1 convolution and 3 × 3 group convolution (GConv). In the NConv3 × 3 convolution structure, the number of channels is first reduced to 1/2 of the input channels by a 1 × 1 convolution, which compresses the feature map and thus reduces the number of parameters. Since the compression of the feature map causes the loss of effective keypoint information, 3 × 3 GConv is then applied to divide the input feature map into g groups by channel; each convolution kernel is divided into groups accordingly, and each group convolution operation generates a new feature map to obtain more feature maps and supplement the feature information. Finally, the results of 1 × 1 convolution and 3 × 3 GConv operations are concatenated together from the channel dimension as the new output. The flow of the constructed L-Basicblock structure is shown in Figure 2b.

#### 2.2.2. Comparison of the Number of Parameters and Computations

To demonstrate the effectiveness of the proposed lightweight residual module L-Basicblock in reducing the number of parameters and computations, the number of parameters and computations are calculated using the formula and compared with the original Basicblock residual module.

Suppose that the input image $I\in {\mathbb{R}}^{W\times H\times C}$ has a feature map with the size $W_{in}\times H_{in}\times C_{in}$ after the first feature extraction stage of HRNet, and the output feature map with the size $W_{out}\times H_{out}\times C_{out}$ is obtained after information processing by feeding this feature map into the Basicblock module. Then, the number of convolutional layer parameters (Params, Parameters, the total number of parameters to be trained in the model) of a Basicblock module can be expressed as follows:

(3) $${\mathrm{Params}}_{B}=k\times k\times C_{in}\times C_{out}+k\times k\times C_{out}\times C_{out}\approx 18{C_{in}}^{2}$$

where *k* is the convolution kernel size, $C_{in}$ is the number of input channels of the first 3 × 3 convolution, and $C_{out}$ is the number of output channels of the first 3 × 3 convolution. $C_{in}$ = $C_{out}$ is assumed here for the convenience of calculation and comparison.

The amount of computation (FLOPs, floating-point operations) for a Basicblock module containing convolutional layers can be approximated as follows:

(4) $${\mathrm{FLOPs}}_{B}\approx 2\times k\times k\times C_{in}\times C_{out}\times W_{out}\times H_{out}\approx 18{C_{in}}^{2}W_{out}H_{out}$$

where *W_out_* is the output width of the Basicblock module, $H_{out}$ is the output height of the Basicblock module, and the output feature map size of both the first 3 × 3 convolution and the second 3 × 3 convolution here is $W_{out}\times H_{out}\times C_{out}$ .

The modified number of parameters is effectively reduced compared with the original Basicblock. The convolutional layer of the L-Basicblock structure consists of two NConv3 × 3, where each NConv3 × 3 contains a 1 × 1 convolution and 3 × 3 GConv. The input size of the 1 × 1 convolution is $W\times H\times C_{in}$ , and the output size is $W\times H\times C_{in}/2$ ; then, the number of parameters of the 1 × 1 convolution is ${C_{in}}^{2}/2$ . The input size of 3 × 3 GConv is $W\times H\times C_{1}$ , and the output size is $W^{\prime }\times H^{\prime }\times C_{2}$ . Then, the number of parameters of 3 × 3 GConv is $9/2\times C_{in}$ . Therefore, the total number of convolution parameters of L-Basicblock is ${C_{in}}^{2}+9C_{in}$ , which is much smaller than the number of convolution parameters of the original Basicblock [10] module $18{C_{in}}^{2}$ .

The computation of the 1 × 1 convolution is ${C_{in}}^{2}/2\times W\times H$ , and the computation of 3 × 3 GConv is $9/2\times C_{in}W_{out}H_{out}$ . The computation of the convolutional layer of the L-Basicblock module is ${C_{in}}^{2}WH+9C_{in}W_{out}H_{out}$ , which is much lower than the computation of the convolutional layer of the original Basicblock module $18C_{in}^{2}W_{out}H_{out}$ . Therefore, the number of parameters and computations of L-Basicblock is greatly reduced compared with the original Basicblock, but the module’s lightweight nature will inevitably cause some information loss and lead to the performance degradation of pose estimation.

### 2.3. EL-HRNet Model Incorporating CBAM Attention Mechanism

**Figure 3:**
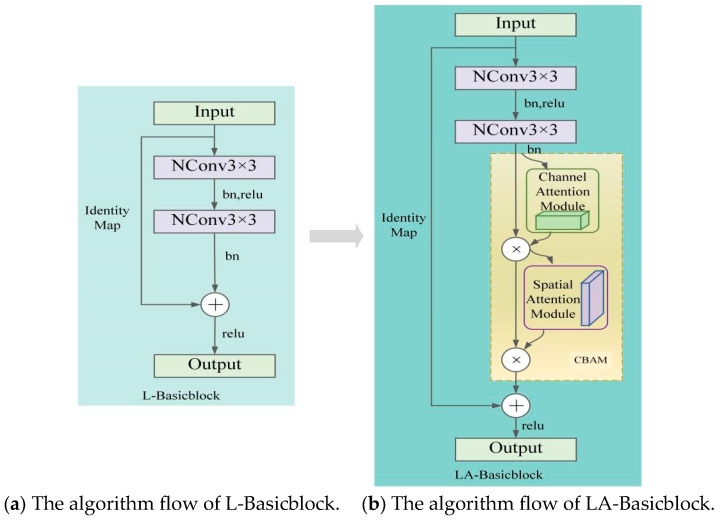
Comparison of L-Basicblock and LA-Basicblock algorithm flow. (**a**) The algorithm flow of L-Basicblock; (**b**) The algorithm flow of LA-Basicblock.

To compensate for the feature information loss in the human pose estimation process brought about by the introduction of L-Basicblock, the LA-Basicblock module shown in Figure 3b is further constructed by introducing a smaller-overhead CBAM attention mechanism into the L-Basicblock structure shown in Figure 3a. This module first extracts information from the feature map of the input module through two NConv 3 × 3 convolution structures to obtain the output feature map and then pays attention to the key channel and spatial information in the output feature map through the CBAM at the same time to obtain rich feature information and improve the performance of pose estimation while maintaining the low number of parameters and computations of the model.

#### 2.3.1. CBAM Attention Mechanism

**Figure 4:**
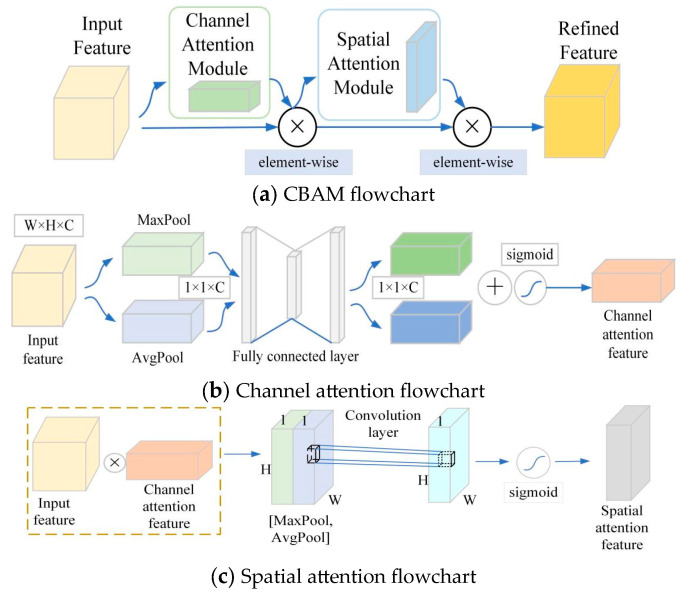
CBAM flowchart. (**a**) CBAM flowchart; (**b**) Channel attention flowchart; (**c**) Spatial attention flowchart.

CBAM is a lightweight, general-purpose module that can be integrated into any convolutional network architecture. It consists of two main parts: the channel attention module and the spatial attention module. The CBAM flowchart is shown in Figure 4a.

The channel attention module uses the channel relationships of features to generate channel attention maps of keypoints, and the detailed structure of the module is shown in Figure 4b. For the input feature map $F\in {\mathbb{R}}^{W\times H\times C}$ of the attention module, the channel attention module is concerned with which information in the input image is meaningful for the output keypoint feature map. To efficiently compute the channel attention, the spatial dimension of the input feature map is compressed. Firstly, two different spatial context features, $F_{c}^{\max}$ and $F_{c}^{avg}$ , are generated using average pooling and maximum pooling operations to aggregate the valid keypoint spatial information in the feature map, and then each of the two features is passed through a shared network to generate a one-dimensional channel attention feature map, $F_{c}\in {\mathbb{R}}^{1\times 1\times C}$ , to aggregate the keypoint information. The shared network contains a perceptron with hidden layers, a multi-layer perceptron (MLP), and the feature size of the hidden layers is set to ${\mathbb{R}}^{1\times 1\times C/r}$ to reduce the parameter overhead, where *r* is the compression ratio. Finally, the channel attention feature map is output by summing and merging at the element level to obtain the valid keypoint channel information. The computation process of the channel attention module is expressed by the following equation:

(5) $$F_{c}=\sigma \left(MLP\left(MaxPool\left(F\right)\right)⊕MLP\left(AvgPool\left(F\right)\right)\right)=\sigma \left(MLP\left(F_{c}^{\max}\right)⊕MLP\left(F_{c}^{avg}\right)\right)$$

where σ represents the sigmoid activation function, *MaxPool* is the maximum pooling layer, *AvgPool* is the average pooling layer, and ⊕ represents the element-by-element summation.

The spatial attention module uses the spatial relationship between features to generate a spatial attention map, which is shown in Figure 4c. It focuses on the information area of “where” the important keypoint of the input feature map is, which is complementary to the channel attention module. First, the channel attention feature map *F_c_* and the original input feature map F are multiplied at the element level to obtain the input feature map of the spatial attention module; then, it is max-pooled and average-pooled along the channel dimension, and the output is concatenated to generate a valid feature descriptor, which can effectively highlight the information regions of keypoints by applying the pooling operation along the channel dimension. A convolutional layer is applied to the concatenated feature descriptor to generate a two-dimensional spatial attention feature map $F_{s}\in {\mathbb{R}}^{W\times H\times 1}$ .

The calculation process of the spatial attention module is expressed in the following equation:

(6) $$F_{s}=\sigma \left(f_{7\times 7}\left(\left[MaxPool\left(F⊗F_{c}\right);AvgPool\left(F⊗F_{c}\right)\right]\right)\right)=\sigma \left(f_{7\times 7}\left(\left[F_{s}^{\max};F_{s}^{avg}\right]\right)\right)$$

where $f_{7\times 7}$ denotes a two-dimensional convolutional layer with a convolutional kernel size of 7, $F_{s}^{\max}\in {\mathbb{R}}^{W\times H\times 1}$ is the spatial feature map with maximum pooling, and $F_{s}^{avg}\in {\mathbb{R}}^{W\times H\times 1}$ is the spatial feature map with average pooling.

The CBAM first aggregates the channel information of human keypoints through a channel attention module, and then it will go through a spatial attention module to obtain the spatial information of relevant keypoints and finally obtain the keypoint feature map $F^{\prime }\in {\mathbb{R}}^{W^{\prime }\times H^{\prime }\times C^{\prime }}$ by weighting to obtain the valid human keypoint information. The arithmetic formula of the attention mechanism is as follows:

(7) $$F^{\prime }=F_{s}⊗\left(F_{c}⊗F\right)$$

where *F* represents the feature map of the input CBAM attention mechanism, $F_{s}$ represents the feature map of the output channel attention module, $F_{c}$ represents the feature map of the output spatial attention module, $F^{\prime }$ represents the feature map of the final output of the CBAM attention mechanism, and ⊗ represents element-by-element multiplication.

#### 2.3.2. EL-HRNet Model Incorporating Attention Mechanism

**Figure 5:**
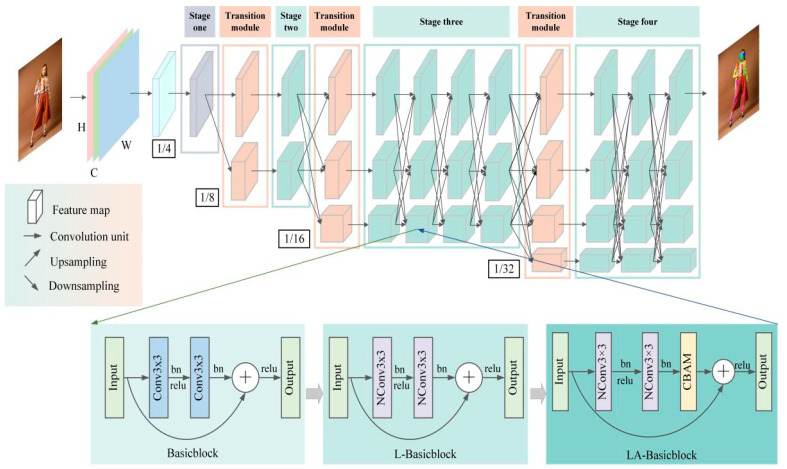
Framework diagram of the improved EL-HRNet model.

After the lightweight improvement of Basicblock in the original HRNet model and the introduction of the CBAM attention mechanism, the effective human keypoint information can be quickly obtained, and a high-quality keypoint heatmap can be output. The framework of the EL-HRNet model with the LA-Basicblock residual module is shown in Figure 5.

Assuming that the input feature of L-Basicblock is $X\in {\mathbb{R}}^{W_{in}\times H_{in}\times C_{in}}$ and the output feature is $Y\in {\mathbb{R}}^{W_{out}\times H_{out}\times C_{out}}$ , the computation process is expressed by the following equation:

(8) Y1=BNfNConv3×3ReLUBNfNConv3×3X

(9) Y=ReLUY1⊕X

where *Y*_1_ denotes the feature map of the intermediate transition, Batch Normalization (*BN*) denotes the batch normalization operation, Rectified Linear Unit (Re*LU*) is the modified linear unit, $f_{\mathrm{NConv}3\times 3}$ denotes the operation of the NConv3 × 3 convolutional structure, and ⊕ denotes the residual connection.

With the introduction of the CBAM attention mechanism, the formula for LA-Basicblock can be expressed as follows:

(10) Y=ReLUSACAY1⊕X

where *CA* denotes the channel attention operation, and *SA* denotes the spatial attention operation.

## 3. Experiment and Discussion

In this study, two large datasets, COCO2017 [27] and MPII [28], were selected according to the HRNet model to train, validate, and test the improved EL-HRNet human pose estimation model. The backbone network is HRNet-32:32 is the width of the first high-resolution branch, and the widths of the other three subnetworks are 64, 128, and 256, respectively.

**Figure 6:**
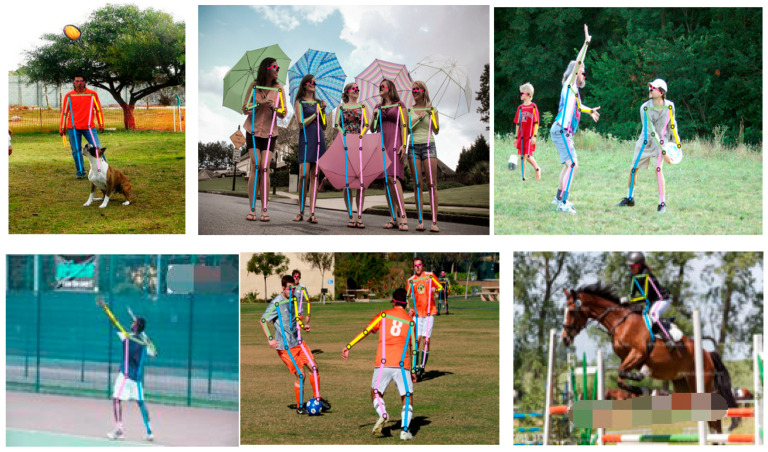
Graph of validation results on the COCO dataset.

Some estimation results of the EL-HRNet model on the COCO2017 dataset are shown in Figure 6.

From the first and second pictures in Figure 6, it can be seen that the proposed model can accurately locate human skeletal points in both single- and multi-person pictures; from the third and fourth pictures, it can be seen that the model can also locate human skeletal points when the human body is seen from the side and the back; from the fifth and sixth pictures, it can also be seen that the model can better identify human skeletal points in complex backgrounds and occlusion situations. Therefore, EL-HRNet can accurately recognize the human body pose in various scenes.

To verify the performance of the model, the experiments also analyzed the Simple Baseline [29], Lightweight [30], ViPNAS [31], Small HRNet [32], Lite-HRNet [32], Hourglass [33], and several other typical lightweight human pose estimation methods, and all methods used the same size input for comparison. The networks and the corresponding backbone networks are shown in Table 1.

### 3.1. Dataset Introduction

#### 3.1.1. COCO2017

The COCO2017 dataset contains 200,000 images with 17 keypoints labeled for each human example in the images. Training was performed on the COCO2017 training set, which contains 57,000 images with 150,000 human examples, and validation and testing were performed on the COCO2017 validation set, which contains 5000 images, and on the test set, which contains 20,000 images, respectively.

The standard evaluation criterion is *OKS*:

(11) $$OKS=\frac{\sum_{i}\exp\left(-d_{i}^{2}/2s^{2}k_{i}^{2}\right)\delta \left(v_{i}>0\right)}{\sum_{i}\delta \left(v_{i}>0\right)}$$

where *s* is the object scale, $k_{i}$ is the constant controlling the decay of each keypoint, $v_{i}$ denotes the visibility of the keypoint, and δ is the function that selects the visible keypoints for calculation.

$d_{i}$ is the Euclidean distance between the detected keypoints and their true values,

(12) $$d_{i}=\sqrt{{\left(x_{1}-x_{2}\right)}^{2}+{\left(y_{1}+y_{2}\right)}^{2}}$$

where *x* and *y* represent the coordinates of the points.

The model performance is characterized by average precision and recall, with the following main metrics: AP50 (AP at OKS = 0.50), AP75, AP (average of AP scores at 10 different locations, OKS = 0.50, 0.55, …, 0.90, 0.95), APM (a metric to evaluate the accuracy of detection of medium-scale objects), APL (a metric to evaluate the accuracy of detection of large-scale objects), and AR (the average recall scores at OKS = 0.50, 0.55, …, 0.90, and 0.95, respectively). 

#### 3.1.2. MPII

The MPII dataset contains images of various types of activities from the real world, with full-body annotation of the human body in each image. The dataset contains 25,000 images with 40,000 human instances, of which the test set contains 12,000 human instances, and the rest are all in the training set.

Evaluation criterion: The PCKh metric is used to judge the performance of the model, i.e., the head-normalized probability of correct keypoints, and a keypoint is correctly detected if the position of the detected keypoint falls within a specified threshold. The calculation formula is as follows:

(13) $$\mathrm{PCKh}=\frac{\sum_{p}\delta \left(\frac{d_{pi}}{d_{p}^{h}}\leq T_{k}\right)}{\sum_{p}1}$$

where *p* denotes the *p*-th person, *i* denotes the *i*-th keypoint, *d_pi_* denotes the Euclidean distance between the predicted value and the true value of the *i*-th keypoint of the *p*-th person, $d_{p}^{h}$ denotes the head scale factor of the *p*-th person, i.e., the current head diameter of the person (60% of the Euclidean distance between the upper left point and the lower right point of the rectangular box of the head) is used as the scale factor, *T* is an artificially set threshold, *k* denotes the *k*-th threshold, and if the bracketed condition holds, then *δ* is 1; otherwise, it is 0. The metrics used in this paper are PCKh@0.5 (PCKh value at *T_k_* = 0.5).

### 3.2. Experimental Setup and Dataset Results

In this section, the experimental settings and results on the COCO2017 dataset and MPII dataset are presented. The results are compared with those of other network models to illustrate the validity of our model.

Training setup in COCO2017: The human detection box was expanded to a height-to-width ratio of 4:3, and then the box was cropped from the image and resized to 256 × 192. Data enhancement included random rotation ([−45°, 45°]), random scale transformation ([0.65, 1.35]), and flipping. The model was trained on an NVIDIA 1050Ti graphics card with 4G memory, with the batch size set to 16 and using the Adam optimizer; the initial learning rate was set to 0.001 and dropped by a factor of 10 in epochs 120, 170, 200, and 260, for a total of 300 training epochs.

Training setup in MPII: To compare with other methods, the input size was cropped to size 256 × 256. The network was trained using an NVIDIA 1050Ti graphics card with 4 G memory and a batch size of 8. The total number of training epochs was 210, and the initial learning rate was 0.001, which was reduced by a factor of 10 at epochs 170 and 200. The test procedure used the provided human detection box instead of the detected human detection box.

#### 3.2.1. COCO2017 Dataset Validation and Test Results

The EL-HRNet human pose estimation model proposed in this paper was compared with other lightweight models in terms of the number of parameters, computation, and AP and AR accuracy metrics. As shown in Table 2, on the COCO2017 validation set, the EL-HRNet model result is 0.6% higher than that of ViPNAS [30] in the medium-scale human detection metric APM and slightly (by 0.7%) lower than that of the ViPNAS method in AP metrics; although the number of parameters is 2.2 M more than ViPNAS and the amount of computation is 1.31 G more, the model still achieves a better balance between complexity and accuracy. Except for the Lite-HRNet method with Lite-HRNet-30 as the backbone network and the ViPNAS method, the other lightweight models have a slight advantage in the number of parameters and computation volume, but all of their accuracy indexes are lower than those of the model in this paper. Also, using the MobileNetV3 network backbone, the ViPNAS network parameter number and accuracy are excellent, but EL-HRNet is higher in the medium-scale human detection index of accuracy. It is well known that medium-scale human detection in daily and industrial scenarios is more widely used. Moreover, compared with the best-performing ScaleNAS model, our number of parameters is far smaller, and our FLOPS is only a quarter of that of the ScaleNAS. Overall, the experimental results demonstrate that the study of the HRNet network structure still has its significance.

As shown in Table 3, on the COCO2017 test-dev dataset, the accuracy indicators of the proposed model all perform well. Compared with the Lite-HRNet method, with Lite-HRNET-30 as the main backbone network, the indexes of AP, AP50, AP75, APM, APL, and AR in this paper are 1.0%, 0.8%, 0.6%, 1.6%, 0.3%, and 1.6% higher, respectively. The number of parameters is 3.2 M higher, and the calculation amount is 1.69 G higher. Compared with the Hand-Crafted model, although the accuracy is slightly decreased, our model only needs 5.0 M parameters, which is far less than the 34.0 M parameters of the Hand-Crafted model.

#### 3.2.2. MPII Dataset Validation Results

Table 4 shows the comparison between our method and several typical lightweight human posture estimation methods on the MPII validation set. The input of all methods is the same, and their backbone networks are different. The parameters, computation amount, and PCKh@0.5 accuracy index are compared. Compared with the Lite-HRNet method with the Lite-HRNet-30 backbone network, our method led to increases of 0.4%, 0.1%, 0.6%, 1.6%, 1.1%, 0.4%, and 0.9%, respectively, in the PCKh@0.5 indexes of the head, shoulder, elbow, wrist, hip, knee, and ankle. The average PCKh@0.5mean increased by 0.7%, while the number of parameters was only 3.2 M higher, and the computation amount was only 2.18 G higher. Compared with the Hourglass network model, our model performs better in terms of the average accuracy and parameter number. The average PCKh@0.5mean is increased by 0.2%, while the number of parameters is reduced by 20.1 M. Compared to the best-performing Hourglass + U-Net model, our accuracy performance is not as good, but the number of parameters is 26 M, while the number of our model parameters is only 5.0 M, much lower than the 26 M of the Hourglass + U-Net model. Meanwhile, our calculation quantity is only 2.66 G, which is much smaller than 33.5 G. Therefore, the experimental results show that our proposed model has lower requirements for equipment and computing power, has a higher computational cost, and is more suitable for peripheral devices (e.g., robot control). The experimental results are compared with these models to prove the validity and rationality of the proposed model. In the future, we will continue to optimize the structure of the model to reduce the number of parameters and improve the precision.

For the SimpleBaseline model using the MobileNetV2 or ShuffleNetV2 backbone network, our parameter number and accuracy are more advantageous. For the lightweight model using the MobileNetV3 backbone network, the number of parameters is larger, but the accuracy is higher. For the ViPNAS network, our model still has some room for improvement. For the Hourglass model and the Hand-Crafted model, our parameter numbers have great advantages. For the Lite-HRNet network model, which also adopts the HRNet structure, the accuracy is greatly improved, although the number of parameters is quite large. For the Hourglass + U-Net model and ScaleNAS model, with the best accuracy, our number of parameters and computations greatly reduce the computational cost. These comparative results illustrate the validity and rationality of our modified method. By training, validating, and testing it on the COCO2017 dataset and MPII dataset, the EL-HRNet model is demonstrated to have a good performance in human pose estimation tasks.

## 4. Conclusions

In this paper, a lightweight approach to human pose estimation is presented. Based on the HRNet network, a lightweight and effective human pose estimation model, EL-HRNet, is proposed. Firstly, the Basicblock module is lightweight; specifically, the number of feature graph channels of the Basicblock input is dimensionally reduced by using two-dimensional conventional convolution with a convolution kernel of 1 × 1. Then, the feature graph obtained from dimensionality reduction is obtained by using grouping convolution with a convolution kernel of 3 × 3, thus obtaining the lightweight module L-Basicblock. The CBAM attention mechanism with less overhead is added to L-Basicblock to improve the modeling ability of the channel information and spatial information. Finally, the LA-Basicblock module is constructed. The EL-HRNet model in this paper maintains the information interaction ability of the original network between different channels but reduces the parameter complexity of the Basicblock model. At the same time, it uses an attention mechanism with low computational cost to ensure the accuracy of pose estimation. It is an effective, lightweight human pose estimation model. Although the model in this paper achieves a balance between the complexity and accuracy of human pose estimation, there is still much room for improvement in the accuracy index of the model. Due to the demand for human pose estimation networks on mobile terminals, the number of algorithm parameters and calculations should be considered when estimating the pose on mobile terminals, so a lightweight and accurate model is required. Therefore, the use of the pose estimation model on mobile terminals will be further studied in the future, and how to further improve the prediction accuracy and real-time detection effect of the network model will be studied.

## Figures and Tables

**Table 1:** The networks and their corresponding backbones.

Method	SimpleBaseline [29]	Lightweight [30]	ViPNAS [31]	SmallHRNet [32]	Lite-HRNet [32]	Hourglass [33]	Ours
Backbone	MobileNetV2	MobileNetV3	MobileNetV3	HRNet-W16	Lite-HRNet-30	Stacked Hourglass	EL-HRNet-W32

**Table 2:** Comparison of EL-HRNet with other lightweight human pose estimation models on the COCO2017 validation set.

Method	Backbone	Input Size	#Params	FLOPs	AP (%)	AP^50^ (%)	AP^75^ (%)	AP^M^ (%)	AP^L^ (%)	AR (%)
SimpleBaseline [29]	MobileNetV2	256 × 192	9.6 M	1.59 G	64.6	87.4	72.3	61.1	71.2	70.7
SimpleBaseline [29]	MobileNetV3	256 × 192	8.7 M	1.47 G	65.9	87.8	74.1	62.6	72.2	72.1
SimpleBaseline [29]	ShuffleNetV2	256 × 192	7.6 M	1.37 G	59.9	85.4	66.3	56.5	66.2	66.4
Lightweight [30]	MobileNetV3	256 × 192	3.1 M	0.58 G	65.8	87.7	74.1	62.6	72.4	72.1
ViPNAS [31]	MobileNetV3	256 × 192	2.8 M	0.69 G	67.8	87.2	76.0	64.7	74.0	75.2
SmallHRNet [32]	HRNet-W16	256 × 192	1.3 M	0.54 G	55.2	83.7	62.4	52.3	61.0	62.1
Lite-HRNet [32]	Lite-HRNet-30	256 × 192	1.8 M	0.31 G	67.2	88.0	75.0	64.3	73.1	73.3
Lite-HRNet [32]	Lite-HRNet-18	256 × 192	1.1 M	0.20 G	64.8	86.7	73.0	62.1	70.5	71.2
ScaleNAS [34]	ScaleNet-P2	256 × 192	35.6 M	8.0 G	**75.2**	**90.4**	**82.4**	**71.6**	**81.9**	**80.4**
Ours	EL-HRNet-W32	256 × 192	5.0 M	2.00 G	67.1	86.4	74.2	65.3	72.0	74.9

**Table 3:** Comparison of the effects of EL-HRNet and other lightweight human pose estimation models on the COCO2017 test set.

Method	Backbone	Input Size	#Params	FLOPs	AP (%)	AP^50^ (%)	AP^75^ (%)	AP^M^ (%)	AP^L^ (%)	AR (%)
SimpleBaseline [29]	MobileNetV2	256 × 192	9.6 M	1.59 G	64.1	89.4	71.8	60.8	69.8	70.1
SimpleBaseline [29]	ShuffleNetV2	256 × 192	7.6 M	1.37 G	59.5	87.4	66.0	56.6	64.7	66.0
Lightweight [30]	MobileNetV3	256 × 192	3.1 M	0.58 G	65.3	89.7	73.4	62.6	70.4	71.3
Lite-HRNet [32]	Lite-HRNet-18	256 × 192	1.1 M	0.20 G	63.7	88.6	71.1	61.1	68.6	69.7
Lite-HRNet [32]	Lite-HRNet-30	256 × 192	1.8 M	0.31 G	66.7	88.9	74.9	63.9	71.9	72.7
Hand-Crafted [29]	SBL-50	256 × 192	34.0 M	8.90 G	**70.0**	**90.9**	**77.9**	**66.8**	**75.8**	**75.6**
Ours	EL-HRNet-W32	256 × 192	5.0 M	2.00 G	67.7	89.7	75.5	65.5	72.2	74.4

**Table 4:** Comparison of the effects of EL-HRNet and other lightweight human pose estimation models on the MPII validation set.

	PCKh@0.5 (%)										
Method	Backbone	#Params	FLOPs	Head	Shoulder	Elbow	Wrist	Hip	Knee	Ankle	Mean
SimpleBaseline [29]	MobileNetV2	9.6 M	2.12 G	95.3	93.5	85.8	78.5	85.9	79.3	74.4	85.4
SimpleBaseline [29]	ShuffleNetV2	7.6 M	1.83 G	94.6	92.4	83.0	75.6	82.8	75.9	69.2	82.8
Lightweight [30]	MobileNetV3	3.1 M	0.77 G	95.6	93.9	85.1	79.5	86.3	80.4	75.5	85.9
Lite-HRNet [32]	Lite-HRNet-18	1.1 M	0.27 G	96.1	93.7	85.5	79.2	87.0	80.0	75.1	85.9
Lite-HRNet [32]	Lite-HRNet-30	1.8 M	0.42 G	96.3	94.7	87.0	80.6	87.1	82.0	77.0	87.0
Hourglass [33]	Stacked Hourglass	25.1 M	19.1 M	96.5	95.3	88.4	82.5	87.1	83.5	78.3	87.5
Hourglass + U-Net [35]	Hourglass + U-Net	26 M	33.5 G	**98.6**	**97.0**	**93.0**	**89.2**	**91.7**	**88.9**	**86.0**	**92.4**
Ours	EL-HRNet-W32	5.0 M	2.66 G	96.7	94.8	87.6	82.2	88.2	82.4	77.9	87.7

## Data Availability

The data presented in this study are available on request from the corresponding author. The data are not publicly available because, in order to adapt it to our study, we processed the dataset.
